# Interpretable Machine Learning Insights into Adhesion and Modulus of Biomedical HA–Dopamine Hydrogels

**DOI:** 10.3390/gels12030206

**Published:** 2026-02-28

**Authors:** Yuze Zhang, Yabei Xu, Yimin Shi, Daxin Liang

**Affiliations:** 1 Key Laboratory of Bio-Based Material Science and Technology, Ministry of Education, Northeast Forestry University, Harbin 150040, China; 2 Key Laboratory of Science and Engineering for the Multi-Modal Prevention and Control of Major Chronic Diseases, Ministry of Industry and Information Technology, Zhengzhou Research Institute of Harbin Institute of Technology, Zhengzhou 450000, China

**Keywords:** hyaluronic acid, machine learning, biomedical materials

## Abstract

Hyaluronic acid–dopamine (HA-Dopa) hydrogels have emerged as promising adhesive biomaterials for biomedical applications. However, the complex dependencies between formulation parameters and hydrogel performance pose challenges for rational material design. In this study, an interpretable machine learning framework was developed to investigate the structure–property relationships of HA-Dopa hydrogels. A dataset comprising 228 data points was collected from 37 peer-reviewed publications, representing heterogeneous experimental conditions across different research groups, and gradient boosting regression models were established to predict adhesion strength and elastic modulus, achieving test R^2^ of 0.99 and 0.94, respectively, with stable performance across cross-validation splits. SHAP analysis revealed that HA molecular weight and dopamine substitution degree are the dominant factors governing adhesion, while mechanical properties exhibit more distributed dependence on multiple formulation parameters. The identified synergistic interactions between key features provide potential guidance for targeted formulation optimization. This work demonstrates the utility of interpretable machine learning in elucidating structure–property relationships and accelerating the development of functional hydrogels for biomedical applications.

## 1. Introduction

Hydrogels, as three-dimensional crosslinked polymer networks with high water content, have attracted considerable attention in biomedical fields, including wound dressings, tissue engineering, and drug delivery systems [1,2,3]. Among various functional hydrogels, adhesive hydrogels that can firmly bond to biological tissues are particularly desirable for clinical applications such as wound closure, tissue sealing, and wearable biosensors [4,5]. Inspired by the remarkable underwater adhesion of marine mussels, catechol-based adhesive hydrogels have emerged as a promising strategy [6,7]. Dopamine, a catechol-containing molecule, can be conjugated to polymer backbones to impart strong adhesion through multiple interactions, including hydrogen bonding, π–π stacking, and metal coordination [8,9,10]. Hyaluronic acid (HA), a natural polysaccharide with excellent biocompatibility, biodegradability, and non-immunogenicity, has been widely used as a matrix for biomedical hydrogels [11]. The combination of HA and dopamine (HA-Dopa) yields hydrogels with both favorable biological properties and tissue adhesion capability, making them attractive candidates for biomedical applications.

However, despite significant progress in HA-Dopa hydrogel research, the current development paradigm still faces several limitations. For biomedical applications such as wound dressings and tissue sealants, adhesive hydrogels must simultaneously satisfy two critical requirements. High adhesion strength is essential to ensure stable bonding to wet tissue surfaces, preventing detachment during body movement or fluid exposure [12,13]. Meanwhile, an appropriate elastic modulus is equally important, as it must match the mechanical properties of target tissues to avoid stress concentration and tissue damage while providing adequate structural support [8,14]. The simultaneous optimization of adhesion and mechanical properties, however, presents a significant challenge due to their complex and often competing dependencies on formulation parameters [15,16]. For instance, Luo et al. developed a near-infrared-responsive HA-Dopa hydrogel dressing incorporating mPDA-DFO@LA nanoparticles for wound healing applications [13]. Zhang et al. constructed a GelMA/dopamine/hyaluronic acid composite hydrogel with stable wet-surface adhesion through Schiff base reaction and photo-crosslinking [17]. While these studies demonstrate the versatility of HA-Dopa hydrogels, they predominantly rely on trial-and-error experimental approaches to balance adhesion and mechanical performance, which is time-consuming and labor-intensive. The adhesion strength and elastic modulus are governed by multiple interrelated factors, including HA concentration, molecular weight, degree of dopamine substitution, crosslinker concentration, pH, and the incorporation of secondary polymers. Increasing crosslinking density may enhance modulus but potentially compromise adhesion by reducing the mobility of catechol groups [13,18,19]. Similarly, higher dopamine substitution may improve adhesion but alter the network structure and mechanical behavior. The complex interplay among these parameters makes it challenging to systematically optimize both properties through conventional experimental methods alone. Furthermore, the existing literature reports scattered data under varying experimental conditions, lacking a unified quantitative framework to guide the rational design of HA-Dopa hydrogels with balanced adhesion and mechanical performance [20,21].

Machine learning (ML) has emerged as an effective approach for predicting material properties and revealing structure–property relationships in complex systems. Unlike conventional experimental methods, ML can efficiently handle high-dimensional parameter spaces and establish quantitative correlations between input features and target properties [22,23,24,25]. Moreover, interpretable ML techniques, such as SHapley Additive exPlanations (SHAP) and feature importance analysis, enable the extraction of mechanistic insights from predictive models, addressing the “black box” limitation of traditional ML algorithms [26]. These approaches have been successfully applied in various materials science domains. For instance, ML has been used to predict the electrochemical performance of biomass-derived carbon electrodes in alkali-ion batteries, identifying key structural descriptors governing capacity and stability [27]. Interpretable ML has also been applied to elucidate adsorption mechanisms of microplastics on biomass composites, revealing dominant factors that control adsorption behavior [28]. However, despite extensive experimental studies on HA-Dopa hydrogels, the literature data remain scattered across individual publications with considerable heterogeneity. Reported adhesion strengths range from ~10 kPa to over 90 kPa depending on formulation parameters and testing conditions, complicating direct comparison and systematic understanding of structure–property relationships [29,30,31]. A unified quantitative framework that consolidates cross-study results and enables predictive modeling for HA-Dopa systems is currently lacking [32]. While machine learning has been increasingly applied to hydrogel design, its application to catechol-based HA systems remains unexplored. Given the multi-parameter nature of hydrogel formulation, ML-driven analysis offers a promising approach for deciphering the structure–property relationships in this system [26].

In this study, an interpretable machine learning framework was developed to elucidate the structure–property relationships of HA-Dopa hydrogels for biomedical applications. A comprehensive dataset was systematically constructed from peer-reviewed publications through the Web of Science Core Collection, encompassing key formulation parameters and performance metrics including adhesion strength and elastic modulus. Multiple machine learning algorithms were evaluated for their predictive capability and generalization performance, and the optimal model was identified through systematic hyperparameter optimization. SHAP analysis was employed to quantify individual feature contributions and investigate synergistic interactions among formulation variables, providing mechanistic insights into the distinct factors governing adhesion and mechanical properties. This work demonstrates a data-driven approach for understanding the complex multi-parameter dependencies in HA-Dopa hydrogel systems and offers potential guidance for rational formulation design in biomedical applications.

## 2. Results and Discussion

### 2.1. Dataset Construction and Feature Analysis

A comprehensive dataset comprising 228 data points was collected from 37 peer-reviewed publications. The dataset encompasses six numerical input features, namely HA concentration (HA_C), HA molecular weight (HA_MW), degree of dopamine substitution (DS), crosslinker concentration (CL_C), pH, and secondary polymer concentration (SP_C), along with one categorical feature of secondary polymer type (SP_Type). The two output variables are adhesion strength (AS) and elastic modulus (EM).

**Figure 1:**
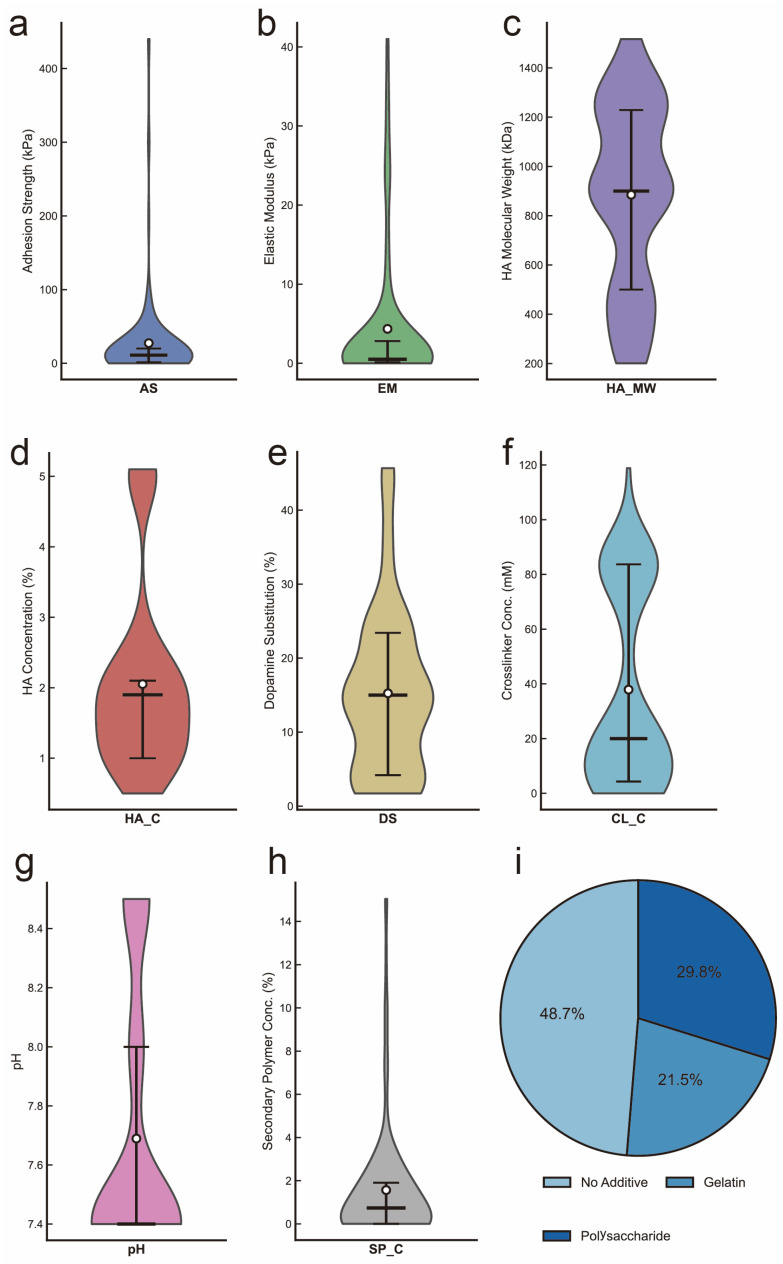
Violin plots of output variables and input features. (**a**) AS. (**b**) EM. (**c**) HA_MW. (**d**) HA_C. (**e**) DS. (**f**) CL_C. (**g**) pH. (**h**) SP_C. (**i**) Distribution of SP_Type categories.

The distribution characteristics of all variables were visualized using violin plots (Figure 1). AS values ranged from approximately 0 to 400 kPa with the majority concentrated below 100 kPa (Figure 1a), displaying a right-skewed pattern suggesting that achieving high adhesion strength requires specific formulation optimization. EM exhibited a highly skewed distribution with most values below 10 kPa (Figure 1b), while some formulations achieved moduli exceeding 40 kPa. Regarding input features, HA_MW showed a broad distribution ranging from 200 to 1500 kDa (Figure 1c). HA_C was predominantly distributed between 0.5% and 5% (Figure 1b). DS ranged from approximately 5% to 45% (Figure 1e). CL_C displayed a bimodal distribution pattern, suggesting two distinct crosslinking strategies adopted in the literature (Figure 1f). The pH values were concentrated in the range of 7.4 to 8.5 (Figure 1g), consistent with physiological conditions. SP_C showed a right-skewed distribution with most values below 5% (Figure 1h). Additionally, one categorical feature of secondary polymer type (SP_Type) was classified into three categories: No_Additive, Gelatin_based, and Polysaccharide (Figure 1i).

**Figure 2:**
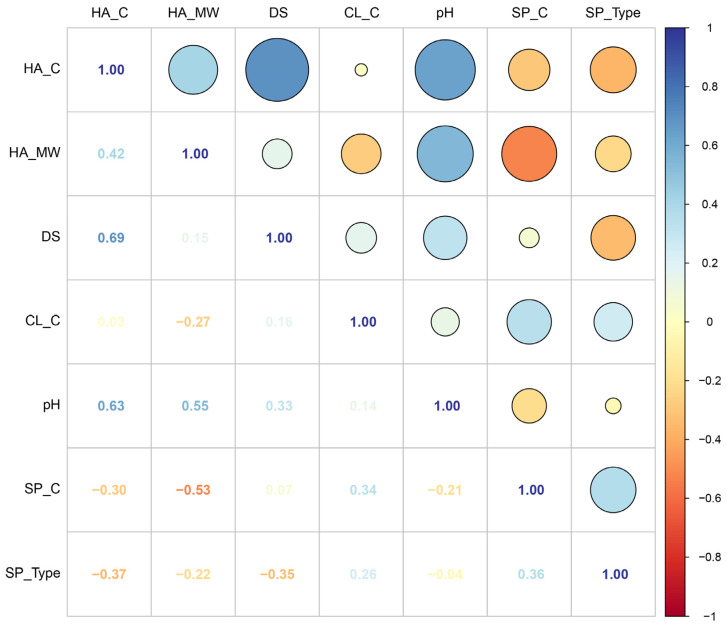
Pearson correlation matrix of input features. The upper triangular matrix displays correlation coefficients as colored circles, where circle size indicates the absolute magnitude of correlation. The lower triangular matrix shows the numerical correlation values. Color scale represents correlation direction and strength: blue indicates positive correlations and red indicates negative correlations.

Pearson correlation analysis was performed to investigate relationships among input features (Figure 2). A strong positive correlation was observed between HA_C and DS (r = 0.69), indicating that higher HA concentrations are often associated with higher dopamine substitution degrees. HA_C also exhibited moderate positive correlations with HA_MW (r = 0.42) and pH (r = 0.63), suggesting that these parameters tend to co-vary in experimental designs.

HA_MW showed a moderate positive correlation with pH (r = 0.55) and a negative correlation with SP_C (r = −0.53), indicating that studies using higher-molecular-weight HA tended to employ lower secondary polymer concentrations. CL_C exhibited weak correlations with most features, suggesting that crosslinker concentration was relatively independently varied across studies. These correlation patterns reflect the inherent complexity of formulation parameters in HA-Dopa hydrogel systems, highlighting the necessity of machine learning approaches to disentangle individual feature contributions.

### 2.2. Model Development and Hyperparameter Optimization

To predict AS and EM of HA-Dopa hydrogels, three representative machine learning algorithms were employed, namely random forest (RF), gradient boosting regression (GBR), and multi-layer perceptron (MLP). RF and GBR are tree-based ensemble methods that have demonstrated superior performance in handling tabular data with complex non-linear relationships. RF constructs multiple decision trees through bootstrap aggregating and averages their predictions to reduce variance, while GBR builds trees sequentially with each tree correcting the errors of its predecessors through gradient descent optimization. These tree-based methods offer inherent advantages including robustness against overfitting, ability to capture feature interactions, and provision of interpretable feature importance metrics. MLP, as a neural network architecture, learns non-linear mappings through multiple hidden layers with non-linear activation functions, offering high model flexibility but typically requiring larger datasets to achieve optimal performance and being more susceptible to overfitting on medium-sized datasets [33,34]. The dataset was randomly split into training and test sets with a ratio of 80:20, and model performance was evaluated using R^2^ and RMSE.

**Figure 3:**
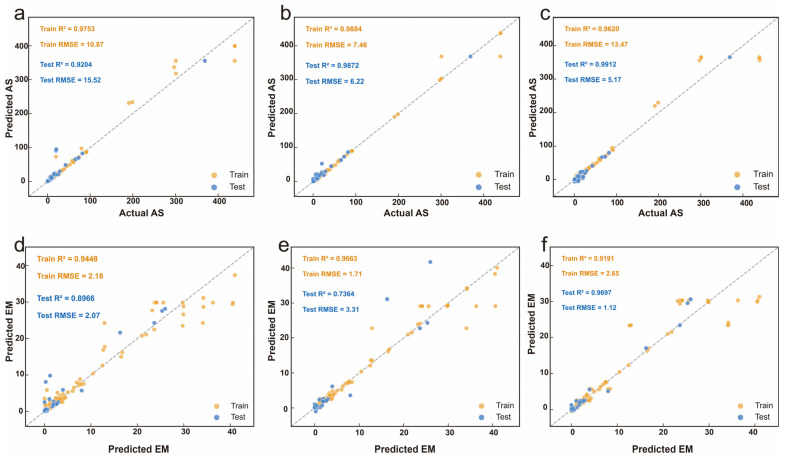
Prediction performance of RF, GBR, and MLP for AS (**a**–**c**) and EM (**d**–**f**). Orange and blue points represent training and test data respectively.

The prediction performance of the three models is presented in Figure 3. For AS prediction (Figure 3a–c), all models achieved training R^2^ exceeding 0.96, demonstrating strong fitting capability. RF (Figure 3a) achieved test R^2^ of 0.9204 with RMSE of 15.52 kPa, showing moderate overfitting and tendency to underpredict high AS values due to its averaging mechanism. GBR (Figure 3b) exhibited the most balanced performance with training R^2^ of 0.9884 and test R^2^ of 0.9872 and RMSE of 7.46 kPa and 6.22 kPa respectively. The minimal gap between training and test metrics indicates excellent generalization, with tight clustering around the diagonal across the entire AS range including the challenging high-adhesion region. MLP (Figure 3c) achieved training R^2^ of 0.9620 and test R^2^ of 0.9912, with RMSE of 13.47 kPa and 5.17 kPa. However, the unusual pattern of test R^2^ exceeding training R^2^ does not reflect superior generalization but rather indicates unstable training behavior characteristic of neural networks with insufficient data [35], suggesting that the 228 samples may be inadequate to constrain MLP parameters effectively.

For EM prediction (Figure 3d–f), the models exhibited more varied performance, suggesting that EM is inherently more challenging to predict from the selected input features, possibly due to the complex dependence of mechanical properties on network density, crosslinking efficiency, and polymer chain interactions. RF (Figure 3d) achieved test R^2^ of 0.8966 with RMSE of 2.07 kPa, showing noticeable dispersion in the intermediate modulus range where subtle formulation differences lead to varied mechanical outcomes. GBR (Figure 3e) displayed pronounced overfitting with training R^2^ of 0.9663 but test R^2^ of only 0.7364 and RMSE of 1.71 kPa and 3.31 kPa, indicating that default hyperparameters allowed the model to memorize training patterns without generalizing to unseen samples. MLP (Figure 3f) achieved training R^2^ of 0.9191 and test R^2^ of 0.9697 with RMSE of 1.12 kPa, again exhibiting the unstable pattern of test performance exceeding training performance. The inconsistent behavior of MLP across both prediction tasks confirms its unsuitability for drawing robust conclusions about structure–property relationships on this dataset size.

Based on the initial model comparison, RF and GBR were selected for hyperparameter optimization due to their interpretability advantages and potential for performance improvement through proper tuning [23,24]. Grid search was employed to systematically explore the hyperparameter space, focusing on two critical parameters controlling model complexity: the number of estimators (n_estimators, ranging from 1 to 128) and maximum tree depth (max_depth, ranging from 1 to 32).

**Figure 4:**
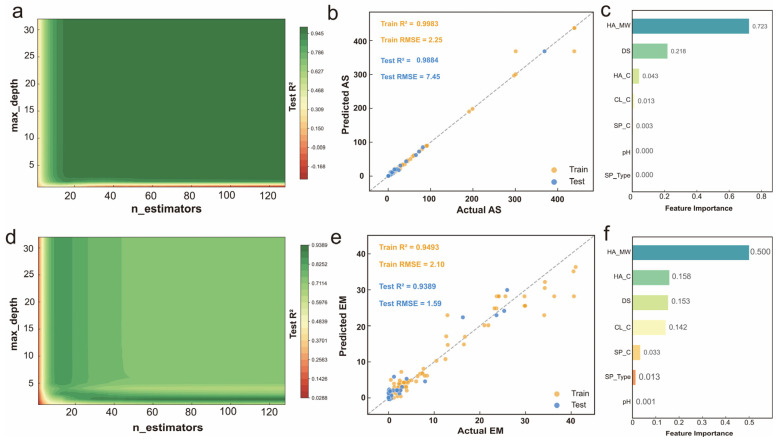
Hyperparameter optimization and feature importance analysis for GBR models. (**a**,**d**) Grid search heatmaps of test R^2^ for AS and EM. (**b**,**e**) Prediction scatter plots of optimized models. (**c**,**f**) Feature importance rankings.

For AS prediction, the surface plot (Figure 4a) reveals that test R^2^ increases rapidly at low parameter values and plateaus at moderate complexity, with the optimal region appearing at intermediate values where model capacity balances learning and generalization. After optimization, the GBR model (Figure 4b) achieved training R^2^ of 0.9983 and test R^2^ of 0.9884, with RMSE of 2.25 kPa and 7.45 kPa respectively, demonstrating tight alignment along the diagonal across the entire AS range. Feature importance analysis (Figure 4c) revealed that HA_MW is the dominant factor with an importance score of 0.723, indicating a strong correlation between HA molecular weight and adhesion strength [4,5,36]. This correlation may be attributed to mechanisms such as extensive chain entanglement at the interface, although the model cannot distinguish between physical entanglement effects and catechol-mediated chemical interactions. DS ranked second (0.218), consistent with the role of catechol groups in mediating adhesion. The combined importance of HA_MW and DS (0.941) dominates AS prediction, while HA_C (0.043), CL_C (0.013), SP_C (0.003), pH (0.000), and SP_Type (0.000) contribute minimally.

For EM prediction, the surface plot (Figure 4d) shows a more sensitive pattern with a narrower optimal region, indicating that EM prediction requires more careful hyperparameter tuning. After optimization, the GBR model (Figure 4e) achieved training R^2^ of 0.9493 and test R^2^ of 0.9389, with RMSE of 2.10 kPa and 1.59 kPa, successfully mitigating the severe overfitting observed in initial models. Feature importance analysis (Figure 4f) revealed a more balanced distribution compared to AS. HA_MW ranked highest (0.500), followed by HA_C (0.158), DS (0.153), and CL_C (0.142), reflecting the multi-factorial nature of mechanical properties. Notably, CL_C showed considerably higher importance for EM than for AS, indicating that crosslinker primarily affects bulk mechanical properties rather than interfacial adhesion [5,37]. These contrasting patterns reveal distinct mechanisms: adhesion is predominantly controlled by HA_MW and DS, while mechanical properties depend on a broader set of compositional factors.

It should be noted that the low feature importance of pH (0.000 for AS, 0.001 for EM) likely reflects the narrow pH range (7.4–8.5) represented in the dataset rather than mechanistic insignificance. Catechol oxidation and quinone formation are known to be strongly pH-dependent, and the limited pH variation in our dataset, which focused on physiological conditions, provides insufficient data to capture these effects.

To further validate model robustness, five-fold cross-validation and multiple random seed experiments were conducted (Tables S1–S6). For the default parameter models, the cross-validation results confirmed that GBR consistently outperformed RF and MLP across different data splits. For the optimized GBR models, the multiple random seed validation demonstrated stable performance with mean R^2^ of 0.9472 ± 0.02 for AS prediction and 0.9234 ± 0.02 for EM prediction. These results confirm that the model performance is reproducible and demonstrate improved stability across different data partitions.

### 2.3. SHAP Analysis and Feature Interactions

To further elucidate the contribution of each feature to model predictions, SHapley Additive exPlanations (SHAP) analysis was performed on the optimized GBR models. Unlike traditional feature importance metrics that only quantify overall contribution magnitude, SHAP provides sample-level explanations that reveal both the direction and magnitude of each feature’s impact on individual predictions, offering deeper mechanistic insights into the structure–property relationships [28].

**Figure 5:**
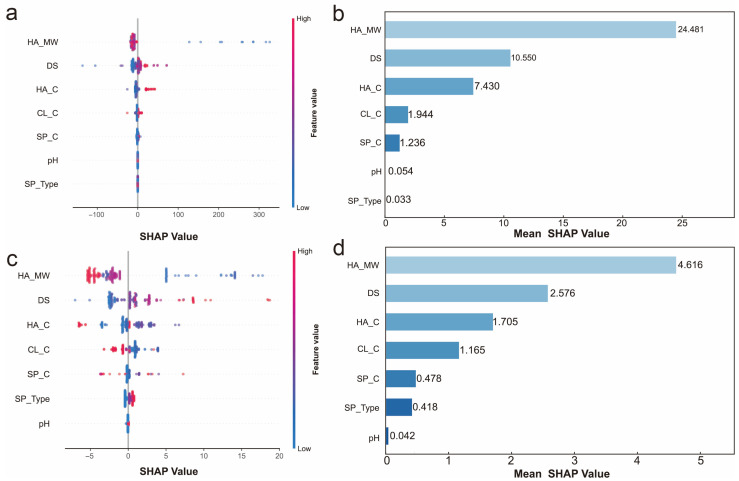
SHAP analysis for AS (**a**,**b**) and EM (**c**,**d**) prediction. (**a**,**c**) Beeswarm plots showing SHAP value distribution for each feature. (**b**,**d**) Mean SHAP value rankings.

The SHAP summary plots and mean SHAP value rankings for AS and EM prediction are presented in Figure 5. For AS prediction (Figure 5a,b), HA_MW exhibited the highest mean SHAP value of 24.481, substantially exceeding all other features and confirming its strong association with adhesion strength. The beeswarm plot (Figure 5a) reveals that high HA_MW values (red points) predominantly contribute positive SHAP values extending up to 300, suggesting that higher molecular weight is associated with enhanced adhesion. Conversely, low HA_MW values (blue points) result in negative SHAP contributions, demonstrating a clear positive correlation between HA_MW and AS. DS ranked second with a mean SHAP value of 10.550, also showing a positive correlation where higher DS values are associated with increased adhesion [2,15]. HA_C ranked third (mean SHAP value 7.430), displaying a more complex pattern with both positive and negative contributions depending on specific sample conditions [3,9,10]. CL_C (1.944), SP_C (1.236), pH (0.054), and SP_Type (0.033) showed minimal contributions, consistent with the feature importance analysis.

For EM prediction (Figure 5c,d), the mean SHAP values were more evenly distributed among features. HA_MW again ranked highest with a mean SHAP value of 4.616, followed by DS (2.576), HA_C (1.705), and CL_C (1.165). The beeswarm plot (Figure 5c) reveals more complex patterns compared to AS prediction. HA_MW shows a clear positive correlation with EM, where high values are associated with positive SHAP values up to 20. DS also exhibits a positive trend, though with greater variability. Notably, CL_C displays a distinct bidirectional pattern, with both high and low values capable of producing positive or negative SHAP contributions depending on other feature combinations, indicating potential interaction effects with other parameters. SP_C (0.478), SP_Type (0.418), and pH (0.042) contributed minimally to EM prediction.

**Figure 6:**
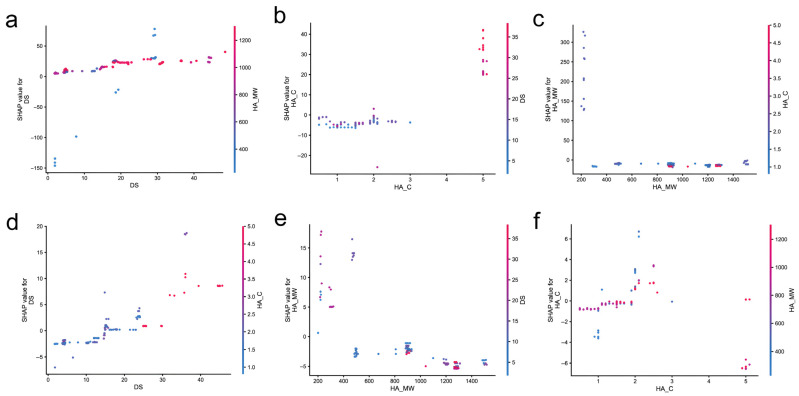
SHAP dependence plots showing feature interactions for AS (**a**–**c**) and EM (**d**–**f**) prediction. (**a**) DS dependence colored by HA_MW. (**b**) HA_C dependence colored by DS. (**c**) HA_MW dependence colored by HA_C. (**d**) DS dependence colored by HA_C. (**e**) HA_MW dependence colored by DS. (**f**) HA_C dependence colored by HA_MW.

To investigate feature interactions, SHAP dependence plots were constructed for the top contributing features (Figure 6). For AS prediction (Figure 6a–c), the DS dependence plot (Figure 6a) colored by HA_MW reveals a non-linear relationship where DS values below 20% show relatively stable and low SHAP contributions, while DS values above 25% exhibit dramatically increased positive contributions, particularly when combined with high HA_MW (red points). This threshold behavior suggests a synergistic effect between DS and HA_MW within the sampled parameter space, where sufficient catechol density combined with long polymer chains is associated with enhanced adhesion. It should be noted that this threshold is a data-driven observation specific to the formulations represented in the literature, rather than a universal physicochemical boundary. Nevertheless, this finding provides practical guidance that, for applications requiring high adhesion strength, formulations targeting DS values above 25% in combination with high-molecular-weight HA (>800 kDa) may be worth exploring [11,38,39]. The HA_C dependence plot (Figure 6b) colored by DS shows that high HA_C values (around 5%) with high DS (red points) produce the largest positive SHAP contributions, while the effect of HA_C alone is moderate without sufficient dopamine substitution. This observation suggests that simply increasing HA concentration without adequate catechol functionalization may not effectively improve adhesion performance [1,17,40]. The HA_MW dependence plot (Figure 6c) colored by HA_C demonstrates that HA_MW values above 800 kDa consistently produce positive SHAP contributions, with the highest contributions observed at HA_MW exceeding 1200 kDa, providing a clear threshold for material selection.

For EM prediction (Figure 6d–f), the DS dependence plot (Figure 6d) colored by HA_C shows a generally positive correlation, with high HA_C (red points) amplifying the positive effect of DS on modulus. This interaction indicates that achieving high modulus requires the combined optimization of both DS and HA_C, as increasing DS alone at low HA_C may not yield significant mechanical enhancement [5,30]. The HA_MW dependence plot (Figure 6e) colored by DS exhibits a more scattered pattern, indicating that the effect of HA_MW on EM is modulated by DS levels, with high DS samples showing greater variability in SHAP values. This variability suggests that the mechanical response to molecular weight changes depends on the crosslinking state of the hydrogel network [41]. The HA_C dependence plot (Figure 6f) colored by HA_MW reveals that high HA_C combined with high HA_MW (red points) produces positive SHAP contributions, while low HA_MW samples show negative contributions regardless of HA_C values. This finding indicates that, for formulations using low-molecular-weight HA, increasing concentration alone cannot compensate for the mechanical disadvantage, and alternative strategies such as enhanced crosslinking or secondary polymer incorporation may be necessary [37,39].

The SHAP analysis reveals predictive relationships that are broadly consistent with polymer physics principles and offers potential guidance for hydrogel formulation design. It should be noted that these relationships represent correlations within the dataset rather than proven causal mechanisms. The dominant positive effect of HA_MW on both AS and EM reflects the role of chain length in forming entangled networks and increasing contact area for adhesive interactions. The synergistic interaction between DS and HA_MW for AS prediction can be rationalized by the cooperative mechanism of catechol-mediated adhesion, where longer chains provide more catechol groups at the interface while maintaining sufficient chain mobility for surface adaptation [41,42,43]. The more balanced feature contributions for EM prediction reflect the multi-factorial nature of mechanical properties, which depend on network density, crosslink efficiency, and chain entanglement collectively [15]. Experimental validation would be required to confirm these mechanistic interpretations.

These findings may inform future formulation strategies for HA-Dopa hydrogels. For applications where adhesion strength is a primary concern, the observed synergy between HA_MW and DS suggests that exploring combinations of high-molecular-weight HA with elevated dopamine substitution could be a promising direction. For applications requiring both adhesion and mechanical performance, the interplay among multiple parameters (HA_MW, HA_C, DS, and CL_C) revealed by SHAP analysis indicates that a multi-dimensional optimization approach may be more effective than single-parameter tuning. Additionally, the relatively minor influence of pH within the physiological range observed in this study suggests that formulation efforts may be more productively focused on compositional parameters.

## 3. Conclusions

In this study, an interpretable machine learning framework was developed to elucidate the structure–property relationships of HA-Dopa hydrogels for biomedical applications. Through the integration of literature-derived experimental data with gradient boosting regression and SHAP interpretability analysis, the multi-factorial dependencies between formulation parameters and hydrogel performance were systematically investigated. The analysis revealed that HA_MW and DS serve as primary determinants of adhesion strength, whereas elastic modulus exhibits a more distributed dependence on HA_MW, HA_C, DS, and CL_C, reflecting the distinct mechanisms underlying interfacial adhesion and bulk mechanical behavior.

The present work demonstrates that machine learning approaches can effectively complement conventional experimental methodologies by enabling systematic extraction of structure–property correlations from heterogeneous datasets. This data-driven framework facilitates the identification of key formulation variables and their interactions, thereby providing quantitative guidance for targeted experimental optimization.

Future efforts may focus on expanding the dataset to encompass broader compositional ranges and standardized characterization protocols, which would enhance model robustness and transferability. Additionally, hyperparameter optimization was limited to n_estimators and max_depth; further tuning of other parameters such as learning rate and subsample ratio may yield additional improvements. Furthermore, external validation using an independent dataset was not performed due to the limited availability of publications meeting our strict inclusion criteria. Future studies should validate these models with newly published experimental data or prospective experiments. The continued integration of machine learning with experimental workflows is anticipated to contribute to the accelerated development of advanced functional hydrogels for diverse biomedical applications.

## 4. Materials and Methods

### 4.1. Data Collection and Preprocessing

This study utilized the Web of Science Core Collection as the primary data source, employing “hyaluronic acid” and “dopamine” as search keywords to systematically collect experimental data from peer-reviewed literature. The search yielded 441 results as of the search date. Each publication was individually screened to ensure that the hydrogel system was based on HA-Dopa or catechol-modified HA, that quantitative data on adhesion strength and/or elastic modulus were reported, and that key formulation parameters were explicitly described. To ensure data consistency, adhesion strength values were exclusively extracted from lap-shear tests performed on porcine skin substrates. Studies using other testing methods or substrates were excluded. Elastic modulus values were derived from both compression testing and oscillatory rheology (storage modulus G’), as both measurements are commonly used in the hydrogel literature to characterize gel stiffness. It should be noted that these methods probe different mechanical regimes, and this pooling may introduce variability that should be considered when interpreting the model results. All values were standardized to kPa. Articles were excluded if they reported: (1) non-hydrogel materials such as coatings or nanoparticles; (2) injectable formulations for in vivo gelation; (3) incomplete quantitative data; or (4) adhesion measurements using non-lap-shear methods or non-porcine skin substrates. After comprehensive screening and quality assessment, 37 research papers were selected that provided complete formulation parameters and performance testing data for HA-Dopa hydrogels [1,2,3,4,5,8,9,10,11,12,13,14,15,16,17,18,19,20,21,29,30,31,36,37,38,39,40,41,42,43,44,45,46,47,48,49,50].

The collected data encompassed six continuous variables and one categorical variable. The continuous variables included HA concentration (HA_C, %), HA molecular weight (HA_MW, kDa), degree of dopamine substitution (DS, %), crosslinker concentration (CL_C, mM), pH, and secondary polymer concentration (SP_C, %). The categorical variable, secondary polymer type (SP_Type), was classified into three categories: No_Additive (encoded as 0), Gelatin_based (encoded as 1), and Polysaccharide (encoded as 2). This encoding is appropriate for tree-based models, which make decisions through binary splits rather than interpreting numerical order. The two output variables were adhesion strength (AS, kPa) and elastic modulus (EM, kPa). Data points with incomplete feature information were excluded to ensure data integrity. The final dataset comprised 228 data points with 7 input features.

### 4.2. Model Development and Evaluation

The dataset was split into training and testing sets using an 80:20 ratio to evaluate model generalization performance. Three machine learning algorithms were systematically evaluated: random forest (RF), gradient boosting regression (GBR), and multi-layer perceptron (MLP). Tree-based ensemble models were emphasized for their effectiveness in handling mixed-type features and capturing complex non-linear relationships, as well as their inherent interpretability through feature importance metrics.

Based on initial model comparison, RF and GBR were selected for hyperparameter optimization using grid search to maximize predictive accuracy. The optimization focused on two critical parameters controlling model complexity: the number of estimators (n_estimators, ranging from 1 to 128) and maximum tree depth (max_depth, ranging from 1 to 32). Model performance was quantified using root mean squared error (RMSE) and coefficient of determination (R^2^). RMSE measures prediction error magnitude and R^2^ quantifies the proportion of variance explained by the model. The RMSE and R^2^ were calculated using Equations (1) and (2):

(1) $$RMSE=\sqrt{\frac{\sum_{i=1}^{n}{\left(y_{t}-y_{p}\right)}^{2}}{n}}$$

(2) $$R^{2}=1-\frac{\sum_{i=1}^{n}{\left(y_{p}-y_{t}\right)}^{2}}{\sum_{i=1}^{n}{\left(y_{t}-y_{m}\right)}^{2}}$$

where y_p_ represents the predicted output value, y_t_ denotes the reported true output value, y_m_ represents the mean of observed output values, n indicates the number of samples in the training or testing datasets.

To evaluate model robustness, five-fold cross-validation was performed with a fixed random state of 42 for the default parameter models. Additionally, multiple random seed validation was conducted using ten different random seeds with 80:20 train–test splitting to assess the stability of model performance across different data partitions.

### 4.3. Interpretability Analysis

SHapley Additive exPlanations (SHAP) analysis was implemented to provide interpretable insights into feature contributions and mechanistic relationships. SHAP values quantify individual feature impacts on predictions based on cooperative game theory, enabling both global feature importance ranking and local explanations of formulation effects on hydrogel properties. SHAP summary plots were generated to visualize the distribution and direction of feature contributions, while SHAP dependence plots were constructed to investigate interactions between key features. This interpretability framework addresses the “black box” limitation of machine learning models and facilitates the extraction of physically meaningful structure–property relationships from the predictive models.

## Data Availability

The dataset supporting this study is publicly available on GitHub at https://github.com/muge223/data (accessed on 16 February 2026).

## Supplementary Materials

The following supporting information can be downloaded at: https://www.mdpi.com/article/10.3390/gels12030206/s1. Table S1: Five-fold cross-validation performance of machine learning models with default hy-perparameters for adhesion strength prediction; Table S2: Model performance stability assessment for adhesion strength prediction using ten different random seeds with 80:20 train–test splitting; Table S3: Five-fold cross-validation performance of machine learning models with default hy-perparameters for elastic modulus prediction; Table S4: Model performance stability assessment for elastic modulus prediction using ten different random seeds with 80:20 train–test splitting; Table S5: Performance stability assessment of the optimized GBR mode for adhesion strength prediction using ten different random seeds; Table S6: Performance stability assessment of the optimized GBR mode for elastic modulus pre-diction using ten different random seeds.
