# Supplementary material for: Interpretable Machine Learning Insights into Adhesion and Modulus of Biomedical HA–Dopamine Hydrogels
Source: Gels. 2026 Feb 28;12(3):206. doi: 10.3390/gels12030206 (PMC13025816; doi:10.3390/gels12030206)
Supplement: Supplementary file 1 [file gels-12-00206-s001.zip › gels-4184743-supplementary.pdf]

Interpretable Machine Learning Insights into Adhesion and Modulus of Biomedical HA—Dopamine Hydrogels

S1. Cross-Validation and Robustness Analysis

Table S1. Five-fold cross-validation performance of machine learning models with default hyperparameters for adhesion strength prediction.

Model	Fold	R^2	RMSE
RF	Fold 1	0.9205	15.5187
RF	Fold 2	0.9671	15.6089
RF	Fold 3	0.9592	2.7726
RF	Fold 4	0.9419	18.2404
RF	Fold 5	0.5186	50.9717
RF	Mean ± Std	0.8614 ± 0.1722	20.6224 ± 16.1031
GBR	Fold 1	0.9872	6.2238
GBR	Fold 2	0.8948	27.9145
GBR	Fold 3	0.9624	2.6617
GBR	Fold 4	0.9111	22.5662
GBR	Fold 5	0.5652	48.4424
GBR	Mean ± Std	0.8641 ± 0.1532	21.5617 ± 16.4706
MLP	Fold 1	-0.2237	60.8713
MLP	Fold 2	-0.0747	89.2064
MLP	Fold 3	-4.8270	33.1190
MLP	Fold 4	-0.1142	79.8897
MLP	Fold 5	-0.0756	76.1865
MLP	Mean ± Std	-1.0630 ± 1.8828	67.8546 ± 19.6239

Table S2. Model performance stability assessment for adhesion strength prediction using ten different random seeds with 80:20 train–test splitting.

Model	Seed	R^2	RMSE
RF	Seed 1	0.9204	15.5237
RF	Seed 2	0.9956	3.6664
RF	Seed 3	0.8528	29.3412
RF	Seed 4	0.9223	21.1379
RF	Seed 5	0.9080	15.7463
RF	Seed 6	0.8225	25.0720
RF	Seed 7	0.9108	18.8679
RF	Seed 8	0.9500	18.9800
RF	Seed 9	0.9335	27.2949
RF	Seed 10	0.9092	9.5011
RF	Mean ± Std	0.9125 ± 0.0454	18.5131 ± 7.5023
GBR	Seed 1	0.9872	6.2238
GBR	Seed 2	0.9770	8.3679
GBR	Seed 3	0.9292	20.3392
GBR	Seed 4	0.8837	25.8583
GBR	Seed 5	0.8403	20.7535
GBR	Seed 6	0.8354	24.1430
GBR	Seed 7	0.8909	20.8689
GBR	Seed 8	0.9035	26.3736
GBR	Seed 9	0.9473	24.3065
GBR	Seed 10	0.9872	3.5713

Model	Seed	R^2	RMSE
GBR	Mean ± Std	0.9182 ± 0.0539	18.0806 ± 8.1868
MLP	Seed 1	-0.2237	60.8713
MLP	Seed 2	-0.2197	60.9515
MLP	Seed 3	-0.0904	79.8458
MLP	Seed 4	-0.1262	80.4629
MLP	Seed 5	-0.2189	57.3278
MLP	Seed 6	-0.1884	64.8656
MLP	Seed 7	-0.2075	69.4341
MLP	Seed 8	-0.1034	89.1886
MLP	Seed 9	-0.0998	111.0306
MLP	Seed10	-0.8706	43.1265
MLP	Mean ± Std	-0.2349 ± 0.2181	71.7105 ± 18.1756

Table S3. Five-fold cross-validation performance of machine learning models with default hyperparameters for elastic modulus prediction.

Model	Fold	R^2	RMSE
RF	Fold 1	0.8682	2.3395
RF	Fold 2	0.4431	5.3491
RF	Fold 3	0.8384	3.8949
RF	Fold 4	0.7901	5.2599
RF	Fold 5	0.8629	2.9513
RF	Mean ± Std	0.7605 ± 0.1611	3.9589 ± 1.2056
GBR	Fold 1	0.7369	3.3051
GBR	Fold 2	0.6321	4.3476
GBR	Fold 3	0.9026	3.0240
GBR	Fold 4	0.8339	4.6789
GBR	Fold 5	0.9411	1.9338
GBR	Mean ± Std	0.8093 ± 0.1125	3.4579 ± 0.9815
MLP	Fold 1	-16.3853	26.8673
MLP	Fold 2	-11.8456	25.6909
MLP	Fold 3	-5.1152	23.9584
MLP	Fold 4	-3.8817	25.3672
MLP	Fold 5	-9.1210	25.3583
MLP	Mean ± Std	-9.2697 ± 4.5515	25.4484 ± 0.9281

Table S4. Model performance stability assessment for elastic modulus prediction using ten different random seeds with 80:20 train–test splitting.

Model	Seed	R^2	RMSE
RF	Seed 1	0.8967	2.0710
RF	Seed 2	0.9569	1.8009
RF	Seed 3	0.8339	4.0625
RF	Seed 4	0.8386	3.5117
RF	Seed 5	0.7927	4.5677
RF	Seed 6	0.7786	4.1983
RF	Seed 7	0.8448	4.3451
RF	Seed 8	0.7076	5.0105
RF	Seed 9	0.7993	4.7618
RF	Seed 10	0.8992	2.0948
RF	Mean ± Std	0.8348 ± 0.0674	3.6424 ± 1.1503
GBR	Seed 1	0.7369	3.3051
GBR	Seed 2	0.9551	1.8384

Model	Seed	R^2	RMSE
GBR	Seed 3	0.8360	4.0375
GBR	Seed 4	0.7935	3.9720
GBR	Seed 5	0.8580	3.7801
GBR	Seed 6	0.7986	4.0045
GBR	Seed 7	0.8646	4.0595
GBR	Seed 8	0.7754	4.3917
GBR	Seed 9	0.8189	4.5234
GBR	Seed 10	0.9045	2.0387
GBR	Mean ± Std	0.8341 ± 0.0609	3.5951 ± 0.8857
MLP	Seed 1	-16.3853	26.8673
MLP	Seed 2	-7.6810	25.5661
MLP	Seed 3	-6.1405	26.6402
MLP	Seed 4	-7.8082	25.9411
MLP	Seed 5	-5.6401	25.8496
MLP	Seed 6	-5.5403	22.8177
MLP	Seed 7	-4.1617	25.0600
MLP	Seed 8	-6.0316	24.5719
MLP	Seed 9	-3.7886	23.2623
MLP	Seed10	-15.9026	27.1237
MLP	Mean ± Std	-7.9080 ± 4.2923	25.3700 ± 1.3862

Table S5. Performance stability assessment of the optimized GBR mode for adhesion strength prediction using ten different random seeds.

Seed	R^2	RMSE
Seed 0	0.8975	28.3168
Seed 2	0.9605	17.6085
Seed 6	0.9343	26.9373
Seed 7	0.9501	20.4156
Seed 8	0.9442	21.4314
Seed 9	0.9448	3.1284
Seed 10	0.9912	2.9521
Seed 12	0.9466	15.7085
Seed 13	0.9449	18.7197
Seed 14	0.9574	18.6747
Mean ± Std	0.9472 ± 0.0221	17.3893 ± 8.0881

Table S6. Performance stability assessment of the optimized GBR mode for elastic modulus prediction using ten different random seeds.

Seed	R^2	RMSE
Seed 0	0.8983	1.9903
Seed 2	0.9056	2.9203
Seed 6	0.9166	2.2684
Seed 7	0.9542	1.4895
Seed 8	0.9385	1.9018
Seed 9	0.9261	2.2096
Seed 10	0.9373	1.6130
Seed 12	0.9544	1.2988
Seed 13	0.9122	2.7298
Seed 14	0.8906	2.4336
Mean ± Std	0.9234 ± 0.0213	2.0855 ± 0.5022
